# Differential hypo-osmotic stress responses and regulatory mechanisms of *Aspergillus sydowii* in amphipod guts and hadal sediments

**DOI:** 10.1128/aem.01448-25

**Published:** 2025-10-29

**Authors:** Zhuo Wang, Yukun Cui, Jiasong Fang, Xi Yu

**Affiliations:** 1 Shanghai Engineering Research Center of Hadal Science and Technology, College of Oceanography and Ecological Science, Shanghai Ocean University 74595, Shanghai, China; Chalmers tekniska hogskola AB, Gothenburg, Sweden

**Keywords:** hadal amphipods, *Aspergillus sydowii*, transcriptomic analyses, osmotic stress, regulation mechanism

## Abstract

Hadal amphipods, which play a vital role in deep-sea ecosystems, harbor gut microbiota that significantly influence host physiology and environmental adaptation. However, understanding of the culturable gut fungi remains limited, particularly their response to the deep-sea osmotic environment. Here, this study reports the successful isolation of *Aspergillus sydowii* XTO612 from the gut of *Hysterocrates gigas*. Comparative physiological profiling with *A. sydowii* DM1, originating from hadal sediment, revealed significant interspecific divergence in hypo-osmotic stress responses (0.1 M NaCl). Low osmolarity conditions were applied as an environmental stressor, and alterations in secondary metabolites, metabolic activity, micromorphology, and reactive oxygen species were observed in the two *A. sydowii* strains under varying osmotic pressures. The results showed enhanced stress responsiveness in *A. sydowii* XTO612. Transcriptomic analyses under hypo-osmotic conditions revealed comprehensive regulatory strategies in *A. sydowii* XTO612, including modulation of membrane permeability, cell wall restructuring, energy metabolism, and osmolyte biosynthesis pathways to optimize osmotic homeostasis. The divergent osmoregulatory strategies in two conspecific marine fungal strains under identical conditions reveal habitat-driven evolution of distinct osmotic regulation mechanisms, highlighting hypo-osmotic stress as a key factor in shaping natural product biosynthesis.

**IMPORTANCE:**

Hadal amphipods play crucial roles in deep-sea ecosystems, yet their gut fungi remain unexplored, representing a major gap in understanding microbial response strategy in extreme environments. While most studies focus on high-osmotic stress, we reveal the unique hypo-osmotic regulatory mechanisms of *Aspergillus sydowii* XTO612, a novel gut-derived strain from hadal amphipods. Comparative analyses demonstrate distinct stress responses, including cell wall remodeling, metabolic reprogramming, and osmolyte biosynthesis, highlighting habitat-driven evolutionary divergence. This study significantly broadens our understanding of fungal osmoregulation, providing groundbreaking insights into fungal regulation under low-osmotic conditions, with potential implications for biotechnology and microbial response strategies in the deep sea.

## INTRODUCTION

The oceans cover 71% of the Earth’s surface, but contain 80% of the planet’s biological resources and are therefore considered to be the origin of life (1). The deep ocean represents a uniquely extreme environment, distinguished by its high pressures, low temperatures, and variable salinity and oxygen concentrations (2). Tectonics and other geological forces have led to diverse seafloor topography, resulting in topographically complex ecological environments such as trenches, seamounts, hydrothermal vents, and cold seeps (3). The presence of a wide range of biological populations in extreme hadal environments is well documented (4, 5). Hadal environments, distinguished by their unique features, necessitate physiological and biochemical adaptations for organisms to thrive and survive within this extreme habitat (6). Amphipods, known for their global distribution and dominant predatory roles in hadal environments (7), have emerged as key subjects in recent studies of adaptation to these extreme habitats (8, 9). The gut microbiota plays a vital beneficial role in defending against pathogens, enhancing the immune system, and improving the host’s adaptability to the environment (10–12), while the composition of the gut microbial community is influenced by host species and environmental factors. A comprehensive investigation into the gut microbiota of hadal amphipods is imperative to enhance the comprehension of their environmental adaptations. Despite the growing recognition of the importance and necessity of gut microbes, the microbial community and diversity within the amphipod gut remain understudied (13). Current research (14–16) on the gut microbiota of hadal amphipods has focused on bacterial biodiversity and biological functions, but there is a gap in the study of fungi isolated from the gut of hadal amphipods.

Fungal organisms must dynamically acclimate to heterogeneous environmental stressors. They achieve this through coordinated morpho-physiological adaptations across various life cycle stages. These stages range from conidial germination to reproductive maturation (17). Hadal fungal endemic to hadal zones must physiologically contend with extreme environments: sustained high hydrostatic pressure, low-temperature conditions, and osmotic gradients. Fungi regulate intracellular water activity in the face of osmotic stress by accumulating compatible solutes, such as glycerol and alginate (18). Under osmotic pressure, fungi adapt to osmotic stress through plasma membrane restructuring (19) and cell wall thickening (20). For example, when exposed to hypersaline conditions, *Aspergillus sydowii* enhances chitin biosynthesis and incorporates α-glucan to create thick, stiff, and hydrophobic cell walls. Such structural rearrangements enable the fungus to adapt to both hypersaline and salt-deprived conditions, providing a robust mechanism for withstanding external stress (21). Additionally, osmotic perturbation induces increased fatty acid unsaturation (19) and morphological alterations in fungal colonies. High osmotic stress induces adaptive strategies in fungi, reallocating resources to dispersal-optimized morphogenesis to colonize new niches. Concurrently, osmotic stress modulates secondary metabolite biosynthesis through stress-responsive regulatory pathways (13, 22).

*A. sydowii* is an extremely widespread fungus found in both terrestrial and marine environments, considered to be a causative agent of coral reef disease (23). As a halophilic fungus prevalent in various marine ecosystems, it has attracted significant research interest due to its ability to produce structurally diverse metabolites (24, 25). In addition, *A. sydowii* can produce a variety of enzymes with significant industrial and biotechnological potential, which is important for the development and use of bioenergy (26, 27). Currently, significant and innovative findings have been made in research on *A. sydowii*’s adaptation to high-salinity environments (28–30). Recent studies (31) have shown that in the halophilic fungus *A. sydowii*, the physiological response to salinity varies under additional stress conditions. Under high osmotic pressure conditions (2.0 M NaCl), *A. sydowii* induces cell wall reorganization and alters the structure and composition of the cell wall by regulating the expression of relevant genes to enhance cellular tolerance to high salt environments (32). Furthermore, it has been found that halophilic fungi can adapt to saturated NaCl conditions by altering the consumption of nutrients in the medium. This adaptation involves metabolic shifts toward non-lipid sources and differences in the production of secondary metabolites (33). Under hyposaline conditions, the alterations in the expression profile are not only driven by the global upregulation of central catabolism and secondary metabolic transferases but are also accompanied by polyol accumulation, demonstrating a coordinated osmoadaptation mechanism to alleviate hypotonic stress (30), while other reactions under low salinity conditions remain to be elucidated. A significant knowledge gap exists in the low-osmotic pressure response and regulation of *A. sydowii*, including the potential divergence of adaptive mechanisms across fungal sources and the regulatory mode of metabolic products.

In our study, to further investigate the regulatory mechanisms of extremophilic fungi, we successfully isolated and characterized *A. sydowii* XTO612, a filamentous fungus derived from the gut microbiota of *Hysterocrates gigas*, and investigated its response to osmotic stress. A comparison was made between the samples and the same species of fungi obtained from three distinctly different locations (strain DM1 was obtained from hadal sediments at 10,898 m depth in the Mariana Trench, strain SDM1 originated from a shallow marine ecosystem, and strain L-5 was isolated from intertidal mudflat samples collected at Luchaogang Beach, China). Two strains of *A. sydowii*, both from hadal environments, were selected with consideration for the differences *in situ* environments. Furthermore, our study conducted a multi-level phenotypic comparison of two *A. sydowii* strains isolated from distinct hadal ecosystems with a comparative assessment. Analyses encompassed metabolic activity, growth rate, micromorphology, and reactive oxygen species (ROS) measurements of the marine filamentous fungus in response to the osmotic stress. Complementary transcriptomic profiling elucidated stress-responsive gene networks governing fungal osmoregulation. These findings establish foundational insights into fungal osmotic-response mechanisms, also suggesting that osmotic variation may drive diversification of fungal secondary metabolism.

## MATERIALS AND METHODS

### Sample collection and isolation, growth rate determination

*H. gigas* specimens were collected from the Challenger Deep of the Mariana Trench in September 2021 using a microbiological pressure-retaining sampler deployed. Two sampling points were located at A (11°19.6098′N, 142°11.283′, 10,895 m) and B (11°22.584′N, 142°35.3102′E, 10,910 m) (14). Amphipods were stored at −80 °C immediately after collection. The samples were transferred to 4°C and rinsed with distilled water. All amphipods were dissected with a sterile scalpel, and the gut tissues were carefully removed and stored in 2 mL centrifuge tubes at 4 °C. The apparatus ensured that there was no external microbial contamination, thus guaranteeing the authenticity of the hadal fungal isolation. The gut contents of the amphipods were diluted to ratios of 10⁻², 10⁻³, and 10⁻⁴ with freshly prepared 3.4 % sterile NaCl solution. Subsequently, 200 µL of each dilution was plated onto potato dextrose agar (PDA) plates, which were then incubated at 28 °C for 4–7 days. Pure cultures were obtained by picking a small amount of mycelium from the colony edge and re-inoculating it into the center of a new plate, based on the methods described in our previous study (28). In addition, three strains of *A. sydowii* from other sources isolated in our laboratory were selected. Strain DM1 was obtained from hadal sediments at 10,898 m depth in the Mariana Trench, strain SDM1 originated from a shallow marine ecosystem, and strain L-5 was isolated from intertidal mudflat samples collected at Luchaogang Beach, China.

Each of the four *A. sydowii* was inoculated onto PDA of different salinities (0 M, 0.1 M, 0.3 M, 0.5 M, 1 M, and 2 M NaCl) incubated at 28°C, and growth diameters were measured daily.

### DNA extraction, PCR amplification, and phylogenetic analysis

All fungal total DNA extracts were taken according to the manufacturer’s instructions of TIAN combi DNA Lyse & Det PCR Kit (TIANGEN BIOTECH, China). Due to insufficient resolution from rRNA gene regions for fungal classification (34), we selected the β-tubulin (*benA*) and calmodulin (*cam*) genes as secondary phylogenetic markers to achieve more confident species-level identification, with all primer sequences detailed in Table S1. The polymerase chain reaction (PCR) was conducted as previously described (28). The PCR amplification products were analyzed by GENEWIZ (Suzhou). The amplified sequences of ITS, *ITS, benA*, and *cam* were submitted to a sequence similarity search by BLASTn against the National Center for Biotechnology Information (NCBI) nucleotide database. The sequences with the highest similarity were selected and trimmed to construct evolutionary trees. Phylogenetic trees were constructed using MEGA11 software with Clustal W (35). The sequences of the entire *ITS*, *benA*, and *cam* genes in *A. sydowii* XTO612 were submitted to the NCBI.

### Morphological characterization of *A. sydowii*

The fungal colony was incubated on PDA medium at a constant temperature of 28°C for 3–7 days. Fresh spore suspension was prepared and drops on 2% agar thin-layer medium (0.1 M: 5.8 g/L NaCl, 20 g/L Agar; 0.5 M: 28 g/L NaCl, 20 g/L Agar) of different salinities, placed on slides and incubated for 2 days, then the morphological characteristics of fungal spore morphology and conidiophore structure were observed using a biological microscope under 40× magnification.

*A. sydowii* spores were scratched from PDA with distilled water. Then, the fresh spore suspension was adjusted to 4 × 10^6^ cells/mL using a hemocytometer. The adjusted spore suspension was placed on PDA medium with different salinities (0 M, 0.1 M, 0.3 M, 0.5 M, 1 M, and 2 M) and incubated at 28°C for 10 days to observe colony morphology.

### Secondary metabolite analysis of two *A. sydowii* strains

Secondary metabolites were extracted according to Xiao (36). Based on preliminary data, a series of salinity gradients (0 M, 0.1 M, 0.3 M, 0.5 M, 1 M, and 2 M) was set for physiological experiments. The most significant differences were observed under the salinity condition of 0.1 M. Therefore, preparation of spore suspensions (4 × 10^6^ cell/mL) and inoculation into potato dextrose broth (PDB) of 0.1 M (low salinity) and 0.5 M salinity (optimal salinity). The fermentation broth was then incubated at 28°C for 10 days in a rotary incubator at 180 rpm. The liquid culture was extracted three times with equal volumes of ethyl acetate. The ethyl acetate crude extract was obtained by concentrating the combined extract using a rotary evaporator. Samples of the crude extracts were prepared by dissolving them in 1 mL of methanol to give a uniform concentration of 100 mg/mL. The solutions were then filtered through a 0.22 µm filter membrane.

The crude extract was analyzed by liquid chromatography-tandem mass spectrometry (LC-MS/MS) at a concentration of 100 mg/mL. Ultra-performance liquid chromatography/tandem mass spectrometry (UPLC-MS/MS) was used as previously described (37) for the qualitative identification of secondary metabolites. The UPLC-MS/MS analysis was performed using a Vanquish UPLC system coupled to a high-resolution mass spectrometer (Thermo Fisher Scientific) equipped with an electrospray ionization interface. The chromatographic separation was performed on a Waters ACQUITY UPLC BEH C18 column (2.1 × 100 mm, 1.7 µm, USA). The mobile phase was delivered at a flow rate of 0.4 mL/min. The column temperature was maintained at 60°C, while the sample injection temperature was set at 10°C. Raw mass spectrometry data were preprocessed using MS-DIAL software to remove noise and extract relevant information such as m/z values and intensities. Relevant features, such as peak area and retention time, were then extracted from the preprocessed data and used to identify and quantify compounds in the sample. The data collected by the mass spectrometer were processed in the form of mass spectrograms using Xcalibur software, which was used to obtain the target compounds.

### Conidial number and evaluation of metabolic activity of polarized growth conidia of *A. sydowii*

Following 4 days of cultivation on PDA under differential NaCl concentration (0.1 M and 0.5 M) at 28°C, *A. sydowii* conidia were aseptically collected through sterile water washing. For the spore suspension obtained, the number of spores was counted under a microscope using a hemocytometer. Three sets of replicates were performed for each set of samples.

The advanced resazurin test, as previously reported, was used to evaluate the metabolic activity of spores subjected to hypotonic treatment (38). Conidial suspensions were prepared by scraping spores from the surface of PDA using 0.1 M and 0.5 M salinity minimal medium (MM: 1% glucose and 70 mM NaNO_3_) at 28°C for 7 days, respectively. The conidia suspensions (4 × 10^6^ cell/mL) were incubated in 200 µL of minimal medium with 0.1 M and 0.5 M at 37°C. After 8 hours, the medium in each well was replaced with 100 µL of medium supplemented with a final concentration of 25 µg/mL resazurin. The plates were then incubated at 37°C for every 12 hours. The assay was performed in a 96-well cell plate in a spectrophotometer (Thermo Scientific Multiskan Spectrum) with an excitation wavelength of 530 nm and an emission wavelength of 590 nm. Three replicate wells were used for each concentration. A negative control was established using the same amount of resazurin and minimal medium to quantify background absorbance.

### Determination of mycelial septum length of two *A. sydowii* strains

Polarized-growth conidia were induced by incubating fresh spores in PDB supplemented with 0.1 M or 0.5 M NaCl under static culture conditions for 18 h. Polarized growth conidia were harvested and stained with a final concentration of 25 µg/mL Calcofluor white (CFW; Sigma-Aldrich) as in the previous report (39) and then incubated in the dark for 5 min at 37°C. Each sample was washed three times with phosphate-buffered saline (PBS). Morphometric analysis of septal spacing was conducted using fluorescence microscopy. Slides were systematically scanned to measure hyphal septum intervals across ≥ 20 randomly selected microscopic fields. All experimental treatments included triplicate biological replicates with independent staining procedures to ensure methodological rigor and data reproducibility.

### Reactive oxygen measurements of *A. sydowii*

ROS measurements were performed as described previously (38). Freshly harvested conidia (4 × 10^6^ cell/mL) were aseptically inoculated into PDB supplemented with 0.1 M and 0.5 M NaCl at 28°C using an orbital shaker at 180 rpm for 60 h in darkness, which is the most representative of the state of mycelial germination under differential NaCl concentration. Subsequently, hyphae were harvested by centrifugation, and DCFH-DA at a final concentration of 10 µM was added into the medium, which was then incubated at 37°C for 1.5 h. After that, the mycelium was rinsed three times with PBS buffer in a 1.5 mL centrifuge tube, and weighed to the same mass and transferred to a new 1.5 mL centrifuge tube, then the mycelium was thoroughly crushed with a pestle and mortar, and the fluorescence intensity was measured by fluorescence microscopy with an excitation wavelength of 488 nm and an emission wavelength of 525 nm. The fluorescence intensity of each sample was measured at various time points, and the heatmap was drawn with the normalized fluorescence value at different times. The experiment was repeated at least three times to ensure the reliability of the results.

### RNA-Seq analysis

To perform RNA sequencing and transcriptomics analysis, the two *A. sydowii* spore suspensions (4 × 10^6^ spores/mL) were inoculated in 0.1 M and 0.5 M NaCl PDB and incubated at 28°C using an orbital shaker at 180 rpm. After 3 days, which corresponds to the logarithmic growth phase and was determined based on previous studies (34), the biomass was harvested. The mycelium was ground thoroughly with liquid nitrogen and stored at −80°C in RNAiso Plus (Takara, Japan).

The RNA extraction, transcriptomics sequencing, and bioinformatics analysis were accomplished by Novogene Co., Ltd (BGI, China). Transcriptome libraries were constructed after end repair, A-tailing, adapter ligation, size selection, amplification, and purification. The final product was loaded on Illumina for RNA sequencing. Gene expression levels were calculated by FPKM values using RSEM software. Differential expression analysis was performed using DESeq2. Q-value ≤ 0.05 and |log_2_(foldchange)|≥1 were set as the threshold for significant differential expression. All differentially expressed genes (DEGs) are annotated against NR, Swiss-Prot (40), Pfam (41), EggNOG (42), GO (43), and KEGG (44) databases.

### qRT-PCR validation

To validate the RNA-Seq results, quantitative reverse transcription PCR (qRT-PCR) analyses were performed as previously described (45). Total RNA was acquired from Novogene Co, Ltd, and RNA reverse transcription was performed according to the manufacturer’s instructions for the Prime Script RT Reagent Kit (Takara, Japan), with all primer sequences detailed in Table S1. Gene expression levels were calculated using the 2^−∆∆Ct^ method (46).

### Statistical analysis

All experiments and experimental groups were performed in at least triplicate, with three biological replicates for each sample. Statistical differences (*P* ≤ 0.05) between the means of spore counts, fluorescence values, and fold changes in marker genes were determined by one-way analysis of variance (ANOVA) and Dunnett’s multiple comparison test. The significant difference for all comparisons was set at *P* < 0.05 (*P < 0.05, **P < 0.01, ***P < 0.001, and ****P < 0.0001). Statistical analysis and graphing with GraphPad Prism 8.0.2.

## RESULTS

### Identification of *A. sydowii* XTO612 from the gut of hadal amphipods

**Fig 1:**
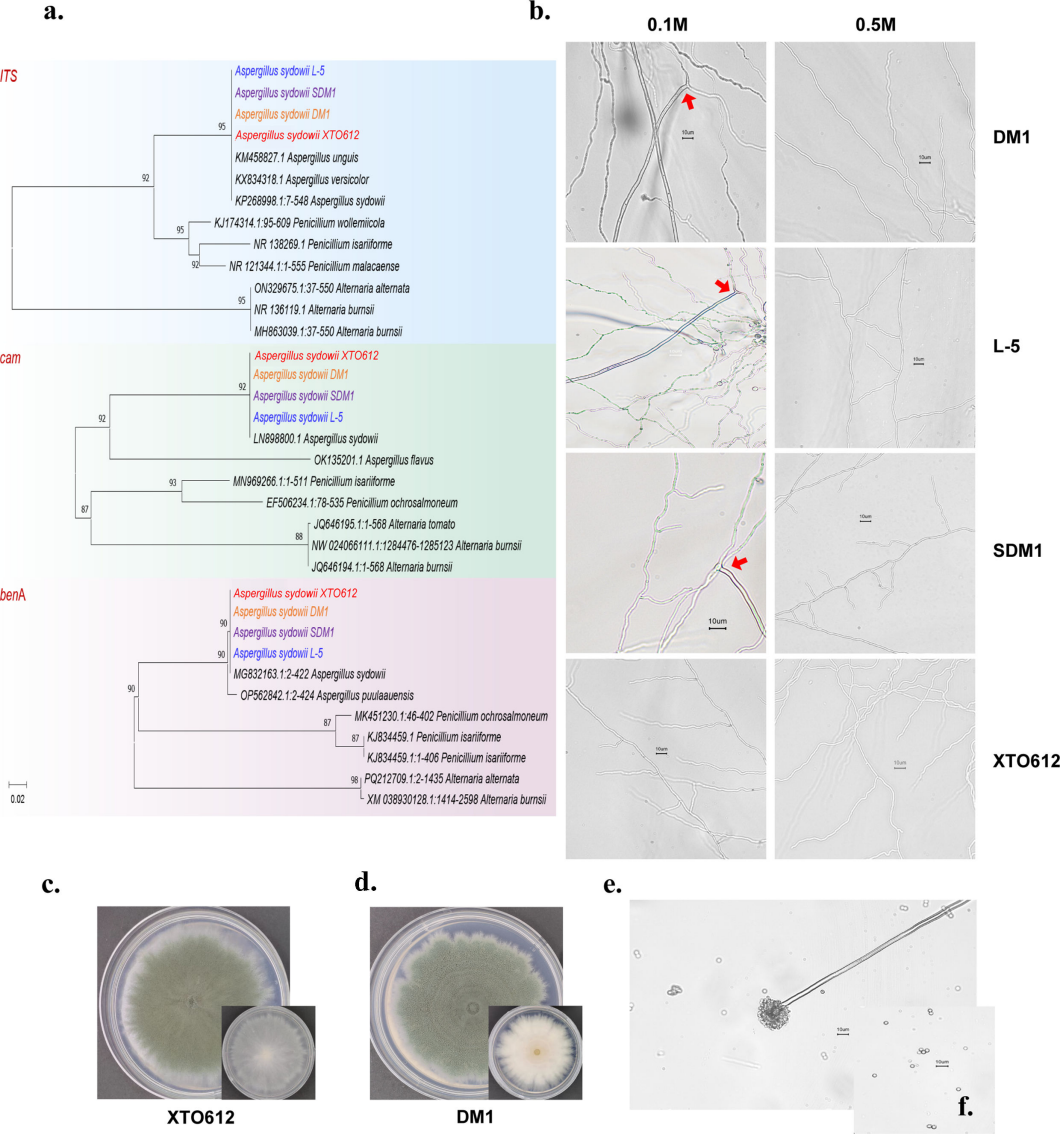
Identification of different sources of *A. sydowii*. (**a**) Phylogenetic tree based on *ITS*, *cam*, and *benA* gene sequences. Bootstrap analysis was performed using 1,000 replicates. (**b**) Mycelial morphology of four strains of *A. sydowii* after 2 days of incubation at 28°C on 0.1 M and 0.5 M salinity agar media. Scale bar represents 10 µm. (**c and d**) Plate morphology of *A. sydowii* originating from the hadal amphipods gut and sediment, respectively, on PDA after 7 days of culture at 28°C. (**e and f**) Conidiophores and conidia on PDA.

*Aspergillus sydowii* XTO612 was isolated and characterized from the gut of hadal amphipods in the Mariana Trench. To elucidate the environmental adaptations of *A. sydowii* from different habitats, we conducted a comparative analysis involving *A. sydowii* XTO612 and three strains isolated from hadal sediments, shallow marine ecosystems, and Luchaogang Beach. Phylogenies were reconstructed for the *ITS*, *cam*, and *benA* gene sequences independently using maximum likelihood (ML), neighbor-joining (NJ), and maximum parsimony (MP) methods. The resulting trees revealed highly congruent topologies across all three methods and genes for the major branching patterns. Four strains robustly clustered with *Aspergillus sydowii* reference strains in both *ITS*, *cam,* and *benA* phylogenies, confirming its identity as *A. sydowii* (Fig. 1a; Table S1; Fig. S1).

To compare the osmotic stress tolerance of *A. sydowii* from different habitats, we selected four *A. sydowii* strains from diverse habitats to assess osmo-tolerance and determine mycelial morphological variations under osmotic stress (0.1 M and 0.5 M NaCl). The mycelial morphology of the four *A. sydowii* strains under 0.1 M and 0.5 M salinity conditions was observed microscopically on agar plates; hyphal swelling was absent exclusively in *A. sydowii* XTO612, whereas all three *A. sydowii* strains (DM1, SDM1, and L-5) exhibited hyphal swelling under 0.1 M salinity conditions (Fig. 1b). To elucidate the evolution strategies of *A. sydowii* across geographically distinct hadal zones, we selected two hadal-origin strains of similar isolation depths.

After 7 days of incubation at 28°C, the colony morphology of the two *A. sydowii* species on PDA was almost similar to that of green ascomycete colonies and white margin (Fig. 1c and d). The microscopic morphology of *A. sydowii* XTO612 was observed to exhibit the typical phenotype of conidiophores and conidia (Fig. 1e and f). The salt tolerance of *A. sydowii* was evaluated through the measurement of growth diameter on PDA at varying NaCl concentrations, which demonstrated that 0.5 M NaCl was the optimal concentration (Fig. S2).

### Secondary metabolism of *A. sydowii* differs under hypotonic stress in different habitats

**Fig 2:**
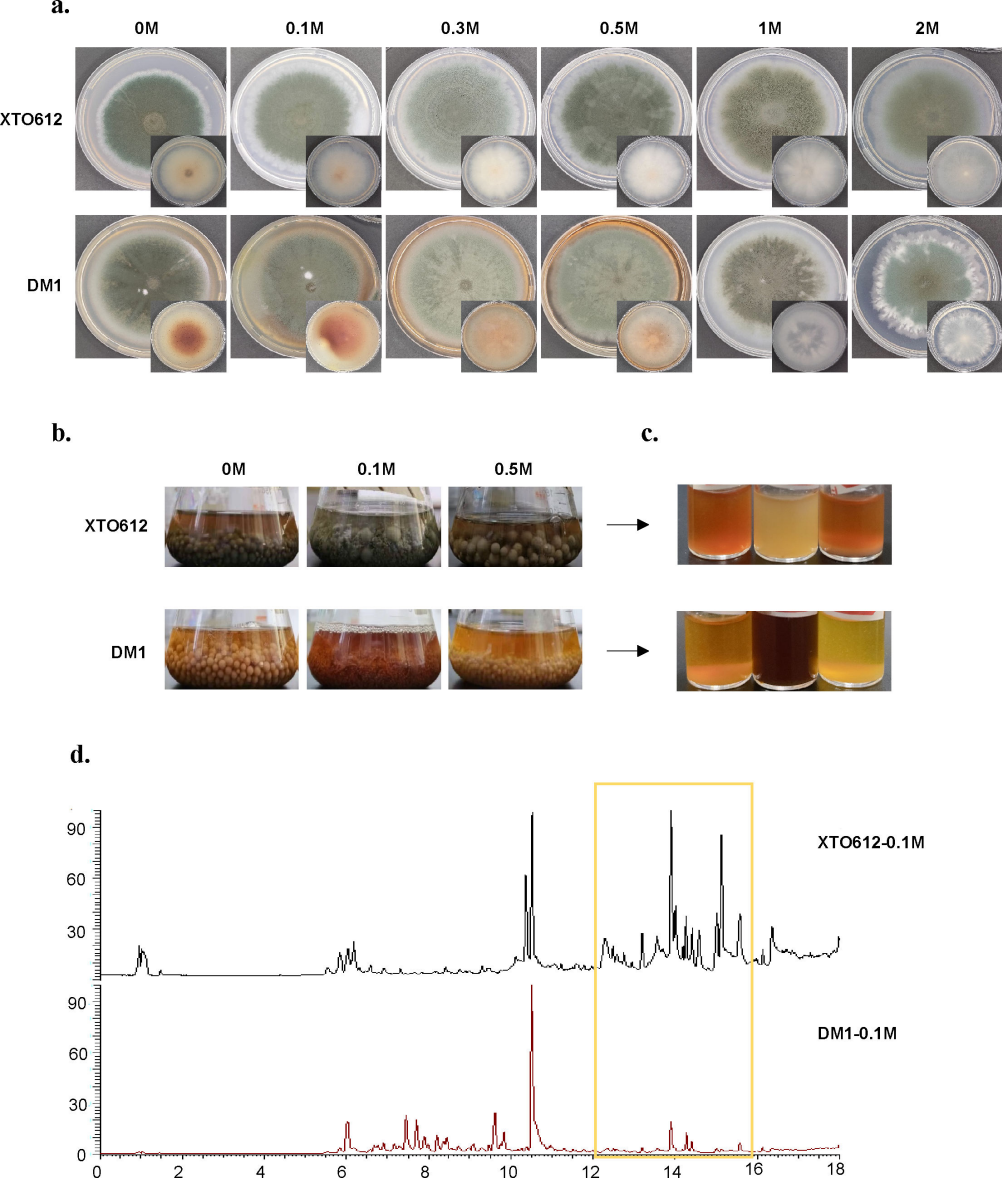
Secondary metabolites from two *A. sydowii* strains. (**a**) Two strains of *A. sydowii* were cultured at 0 M, 0.1 M, 0.3 M, 0.5 M, 1 M, and 2 M salinity conditions for 10 days at 28°C. Colony phenotypes, pigmentation on the back of the colony. (**b and c**) Color changes in the fermentation broth of spores incubated on PDB for 10 days after different salinity treatments (0 M, 0.1 M, and 0.5 M). The fermentation broth and fungal particles of *A. sydowii* DM1 spores accumulated more pigments. (**d**) UPLC-MS/MS spectra of secondary metabolites of two *A. sydowii* strains under 0.1 M salinity conditions.

Fungi that survive in different environments are constantly challenged by a variety of environmental stresses, and the production of secondary metabolites varies with the environment (47). To evaluate the phenotypic variations between the two *A. sydowii* strains derived from distinct habitat sources, the strains were inoculated on PDA with different salinities (0 M, 0.1 M, 0.3 M, 0.5 M, 1 M, and 2 M) and incubated at 28°C for 7 days for observation (Fig. 2a). Our findings reveal significant intraspecific variation in secondary metabolite production between the two *A. sydowii* strains under different salinity conditions. The experimental results demonstrated that salinity exerted a profound influence on pigment biosynthesis, with *A. sydowii* DM1 exhibiting robust pigment production across a range of NaCl concentrations (0 M, 0.1 M, 0.3 M, and 0.5 M NaCl). In contrast, *A. sydowii* XTO612 showed markedly reduced pigmentation capacity at lower salinity levels (0 M and 0.1 M NaCl). Notably, pigment synthesis was completely inhibited in both strains when exposed to elevated salinity conditions (1 M and 2 M NaCl), suggesting a salinity threshold for pigment production in these fungal strains.

Subsequently, fungal suspensions of *A. sydowii* were inoculated in PDB medium with varying salinity levels (0 M, 0.1 M, and 0.5 M NaCl). The results revealed that 0.1 M salinity represented a particularly intriguing inflection point for pigment production, where the responses of the two strains diverged significantly. Notably, *A. sydowii* DM1 exhibited maximal pigment production in the fermentation broth at 0.1 M salinity, whereas *A. sydowii* XTO612 showed the weakest pigmentation under the same condition. This striking contrast highlights the strain-specific modulation of metabolite biosynthesis by salinity (Fig. 2b and c).

To further explore the effects of 0.1 M hypo-osmotic conditions on the two strains, their metabolites were extracted and subjected to UPLC-MS/MS analysis. By comparing the metabolites of the two *A. sydowii* strains under the same hypotonic stress, distinct peaks were found in the total ion chromatogram (TIC) (Fig. 2d). The results showed that the secondary metabolites of the two strains of *A. sydowii* were significantly different at a salinity of 0.1 M NaCl. Subsequently, the mass spectrometry data were subjected to a comparative analysis in the COCONUT, NP Atlas, and StreptomeDB databases (7) to identify the corresponding compounds. To investigate the phenotypic divergence between the two strains under low-osmolarity conditions, 10 compounds exhibiting pronounced alterations in chromatographic signal intensities were identified. The top 10 compounds were identified as A-1 (Unguisin B, C_37_H_56_N_8_O_7_), A-2 (Aspergillicin E, C_39_H_58_N_6_O_9_), A-3 (Radarin B, C_28_H_41_NO_2_), A-4 (Asperorydine N, C_15_H_18_N_2_O_3_), A-5 (Cosmosporaside C, C_34_H_60_O_15_), A-6 (Hormonemate A, C_35_H_62_O_15_), A-7 (2-(ethoxycarbonyl)−4'-carboxydiorcinal, C_18_H_18_O_7_), A-8 (Albatrelin F, C_44_H_58_O_6_), A-9 (18-O-b-d-Glucopyranosyl-18S-hydroxyneodihydroprotolichesterinate21-O-b-d-glucopyranoside, C_33_H_58_O_15_), and A-10 (Calpinactam, C_38_H_57_N_9_O_8_) by database comparison (Table S2).

### Growth capability of *A. sydowii* XTO612 is enhanced under hypo-osmotic stress

**Fig 3:**
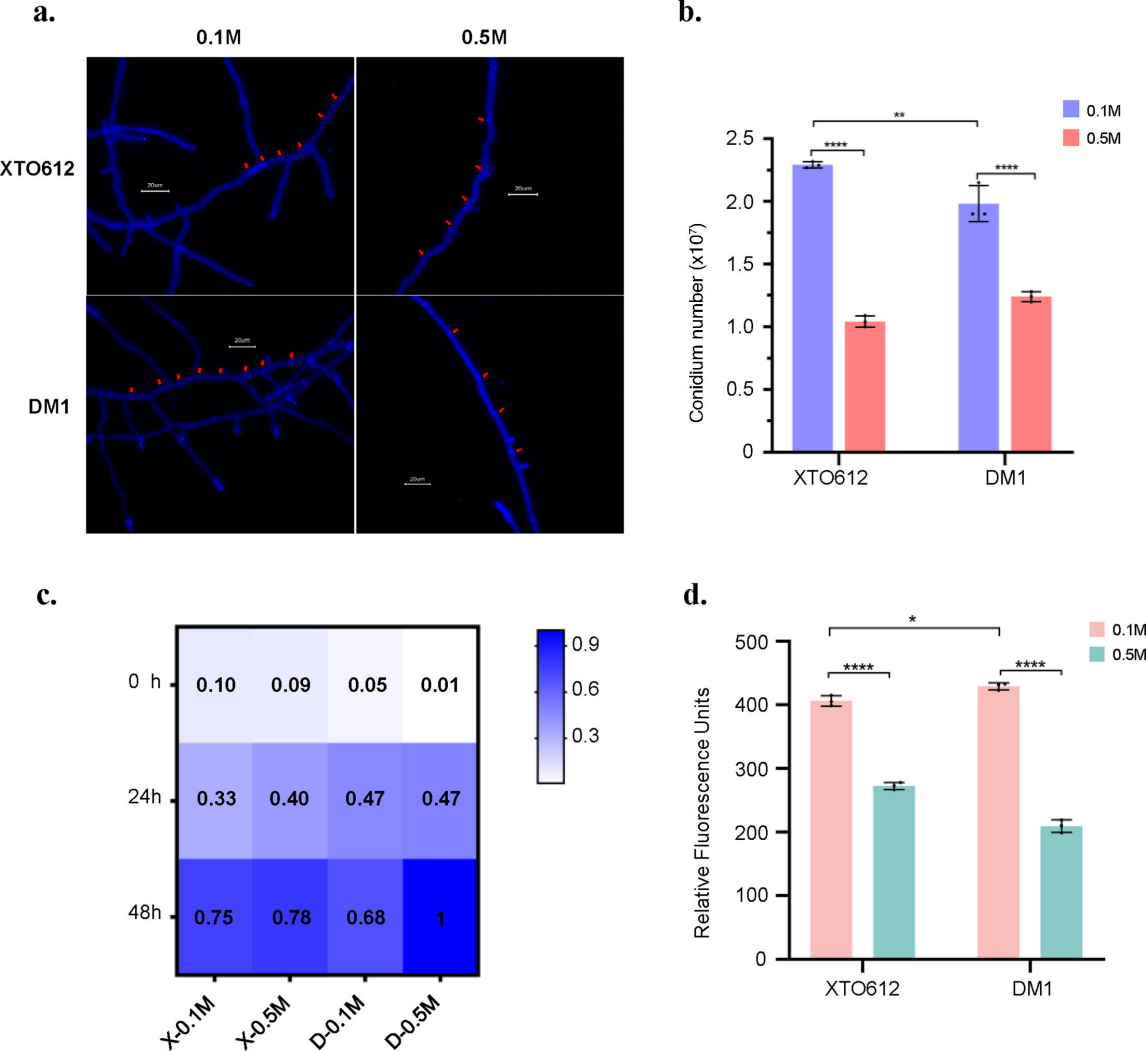
Adaptive adaptation of *A. sydowii* under low osmotic stress. (**a**) *A. sydowii* mycelium stained by CFW under low osmotic stress. The red arrow pointed to the septa stained with fluorochrome. Scale bar represents 10 µm. (**b**) Conidia were inoculated in medium with different salinities (0.1 M and 0.5 M NaCl) and incubated at 28°C for 7 days, then conidia were counted and the determination was repeated three times. (**c**) Conidia were inoculated into medium at different salinities and incubated at 28°C for 8 h. Each well was replaced with 100 mL of medium supplemented with a final concentration of 25 µg/ml of resazurin. Plates were then incubated at 37°C for 12 h each to detect fluorescence values. Each well represents the average of three replicate experiments. The heat map shows the ability of the strain to grow under different salinity conditions. X-0.1 M represents XTO612 at 0.1 M salinity; X-0.5 M represents XTO612 at 0.5 M salinity; D-0.1 M represents DM1 at 0.1 M salinity; D-0.5 M represents DM1 at 0.5 M salinity. (**d**) Fresh spore suspensions were incubated in PDB at different salinities for 60 h after standing incubation in medium containing 10 mM DCFH-DA for 1.5 h at 37°C. ROS production was determined for *A. sydowii* XTO612 and DM1 at 0.1 M and 0.5 M salinity, respectively. Results are the mean of 3 independent experiments, and error bars are standard deviations. Statistical differences (****P < 0.0001 and *P < 0.05) were analyzed by one-way analysis of variance.

Previous experimental validation demonstrated that fresh conidia of *A. sydowii* can reach the polarized growth phase after 10-12 hours of static incubation in PDB medium (47). Following 16 hours of static cultivation in PDB, mycelial morphology was examined via microscopy and CFW staining (Fig. 3a). Quantification of septal length was performed across multiple microscopic fields, with a minimum of 20 independent measurements per replicate and three biological replicates per strain to ensure statistical robustness (Fig. S3). Under 0.5 M NaCl, the mean septal length of *A. sydowii* XTO612 was quantified as 26.48 µm, whereas a statistically significant reduction to 18.65 µm was observed under hypo-osmotic stress (0.1 M NaCl). Similarly, *A. sydowii* DM1 exhibited a septal length of 27.50 µm at 0.5 M NaCl, decreasing to 23.3 µm under 0.1 M NaCl. Comparative analysis revealed that both strains displayed elongated septal intervals under optimal stress relative to hypo-osmotic environments, with *A. sydowii* XTO612 demonstrating a greater reduction in septal length compared to *A. sydowii* DM1 under low-salinity conditions.

Recent studies have demonstrated a significant reduction in conidial production under hyperosmotic conditions (48). To examine whether low-salinity conditions influence conidial production in *A. sydowii* strains from distinct sources, conidial counts for both strains were quantified using a hemocytometer at 0.1 M and 0.5 M NaCl. The results revealed that both strains exhibited enhanced sporulation at 0.1 M salinity compared to 0.5 M salinity. Notably, *A. sydowii* XTO612 displayed a 40% increase in spore production at 0.1 M salinity relative to 0.5 M salinity, whereas *A. sydowii* DM1 showed a 20% increase under the same conditions. Furthermore, *A. sydowii* XTO612 outperformed *A. sydowii* DM1 in spore production under low-osmotic stress, highlighting strain-specific responses to salinity variation (Fig. 3b).

### Metabolic vitality of *A. sydowii* XTO612 is enhanced under hypo-osmotic stress

The transition of spores from dormancy to active growth underscores the adaptability of spores to their environment (34). To assess the difference in spore metabolic activity between the two *A. sydowii* strains under low salinity conditions, we prepared fresh spore suspensions and subjected the spores to low salinity stress and used the fluorescence values of resazurin under a fluorescent enzyme labeler as an indicator for spore metabolic viability assays. Heat maps were plotted based on the values after normalization of the fluorescence values of *A. sydowii* DM1 at 48 h at 0.5 M NaCl (Fig. 3c). The results showed that the metabolic activity of *A. sydowii* XTO612 under low salinity conditions could be restored to that under normal salinity conditions within 48 h. In contrast, the metabolic activity of *A. sydowii* DM1 under low salinity conditions was lower than that of *A. sydowii* DM1 under normal salinity conditions.

Oxidative stress is induced when the level of ROS exceeds the antioxidant capacity of the cell, thus prompting the cell to adopt various defense and repair strategies (49). To further verify whether the oxidative stress responses of the two *A. sydowii* strains differed under hypotonic conditions, a ROS assay kit (Beyotime, China) was employed to detect the intracellular ROS content. The content of ROS was evaluated using fluorescence values. Subsequently, the fluorescence values were normalized to the control group and presented in Fig. 3c to illustrate the relative changes in ROS levels across different treatments. The results showed that both *A. sydowii* exhibited significantly higher ROS levels at 0.1 M salinity compared to 0.5 M salinity (Fig. 3d). The two *A. sydowii* strains exhibited different ROS levels in response to the same salinity stress, with *A. sydowii* XTO612 exhibiting significantly lower ROS levels compared to *A. sydowii* DM1 at 0.1 M salinity.

### Transcriptome overview, DEGs, and enrichment analysis

To clarify the molecular mechanism of regulation of *A. sydowii* in different environmental conditions, especially in low salinity, we conducted RNA sequencing analyses on two *A. sydowii* strains under different osmotic pressure conditions (0.1 M, 0.5 M). Statistical analysis of the FPKM values obtained was conducted to screen for differential genes. Raw reads information was listed in Table S3, which indicated the reliability of RNA-Seq data in this work.

**Fig 4:**
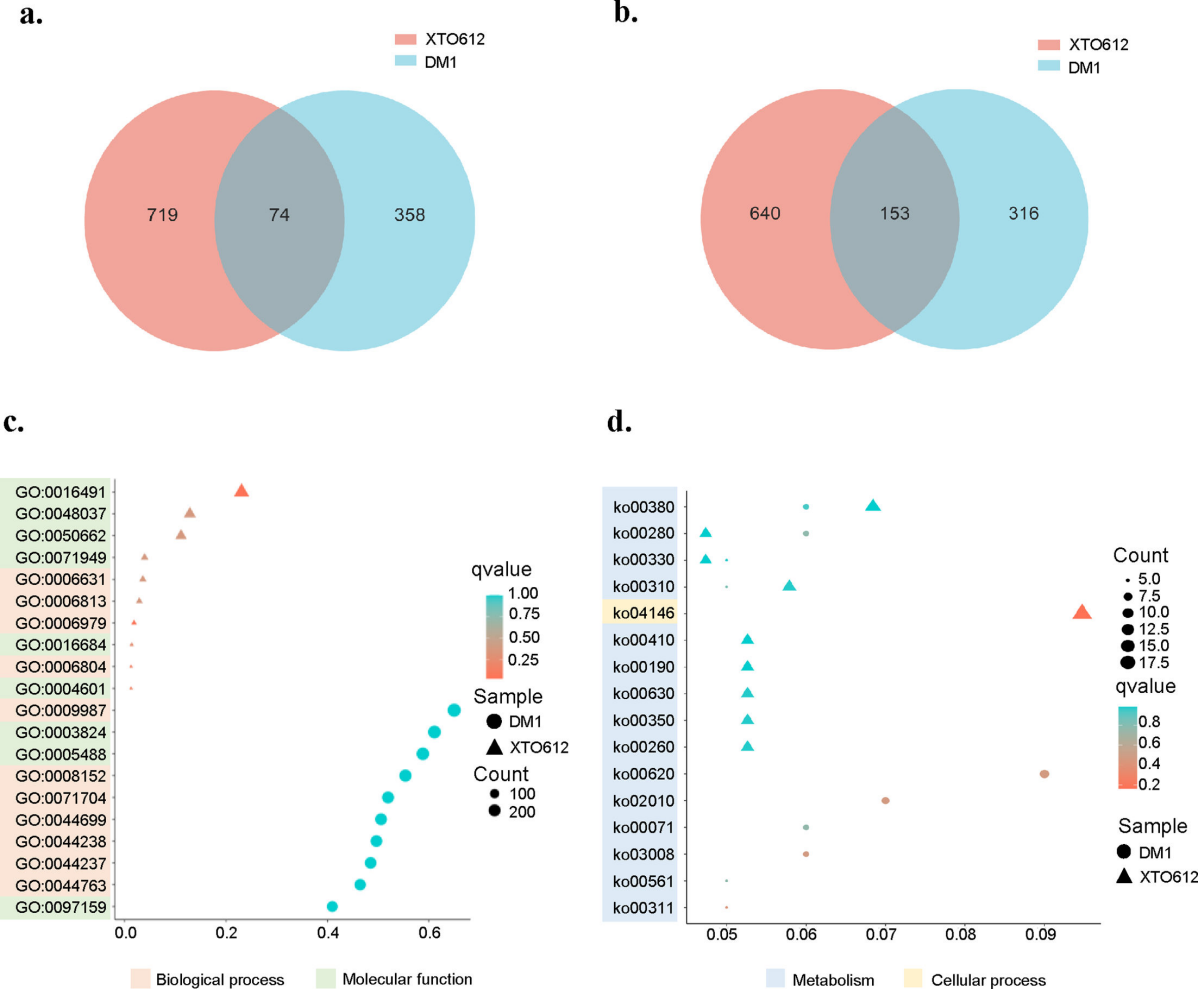
DEG overview of two *A. sydowii* cultured under different salinity (0.1 M and 0.5 M NaCl). (**a**) Differentially upregulated genes under low salinity (0.1 M NaCl) conditions compared to normal salinity (0.5 M NaCl) conditions for both *A. sydowii*. (**b**) Differentially downregulated genes under low salinity conditions compared to normal salinity conditions for both *A. sydowii*. (**c**) Top 10 terms in GO enriched from commonly shared genes of *A. sydowii* XTO612 and DM1 at 0.1 M salinity. (**d**) Top 10 terms in KEGG enriched from commonly shared genes of *A. sydowii* XTO612 and DM1 at 0.1 M salinity. The figure was drawn using OECloud tools.

Principal component analysis of the expression profiles of all samples revealed that the expression profiles of the two *A. sydowii* strains under osmotic stress exhibited statistically significant differences between individuals. A total of 10,270 DEGs were identified in *A. sydowii* XTO612 under 0.1 M and 0.5 M salinity conditions, including 793 upregulated genes and 793 downregulated genes. A total of 902 DEGs were identified in *A. sydowii* DM1 under 0.1 M and 0.5 M salinity conditions, including 432 upregulated genes and 470 downregulated genes (Fig. S4). The focus was on the DEGs of the two strains in relation to the low salinity conditions, and these DEGs were analyzed comparatively. The two strains of *A. sydowii* were further screened for upregulated and downregulated DEGs. The results revealed that *A. sydowii* XTO612 exhibited 719 unique upregulated genes and 640 unique downregulated genes, while *A. sydowii* DM1 displayed 358 unique upregulated genes and 316 unique downregulated genes (Fig. 4a and b). These differential genes may indicate their potential function under low osmotic conditions, which, in turn, may reveal the mechanism of adaptation of the two strains. Further analysis is required to confirm this hypothesis. Gene names and the proteins they encode are listed in Table S4.

DEGs for the two *A. sydowii* strains at 0.1 M salinity were enriched in the KEGG and GO databases. Additionally, the top 10 items in all groups were further enriched in Fig. 4c and d. Comparative transcriptomic analysis revealed that *A. sydowii* DM1 exhibited a paucity of DEGs, with no significant functional enrichment detected in the GO database or KEGG pathways. Consequently, subsequent investigations were prioritized to elucidate the transcriptional dynamics and response mechanisms of *A. sydowii* XTO612 under low-osmolarity stress. Among the top 10 entries analyzed by GO enrichment, two functional categories, cellular components and molecular functions, were mainly involved. Among them, DEGs related to biological processes (GO:0009987) accounted for most of the entries, which were mainly enriched in the GO functional categories oxidoreductase activity (GO:0016491), cofactor binding (GO:0048037), and coenzyme binding (GO:0050662). In the KEGG enrichment analysis, there was a high percentage of DEGs in the pathways of peroxisome metabolism (ko04146); tryptophan metabolism (ko00380); lysine degradation (ko00310); glyoxylate and dicarboxylic acid metabolism (ko00310); and glycine, serine, and threonine metabolism (ko00260). These results implied that the biosynthesis of amino acids and lipid metabolism play an essential role in *A. sydowii* XTO612 under low-osmotic conditions.

The expression patterns of all DEGs identified by qRT-PCR were found to be consistent with those observed in the RNA-seq data, which serves to validate the reliability of the RNA-seq data (Fig. S5). The results substantiated the reliability of the RNA-Seq data and simultaneously demonstrated that transcriptome analysis is a reliable tool for investigating the regulatory mechanisms of filamentous fungi under low osmotic stress conditions.

### Cell wall composition shows variations under hypotonic stress

**Fig 5:**
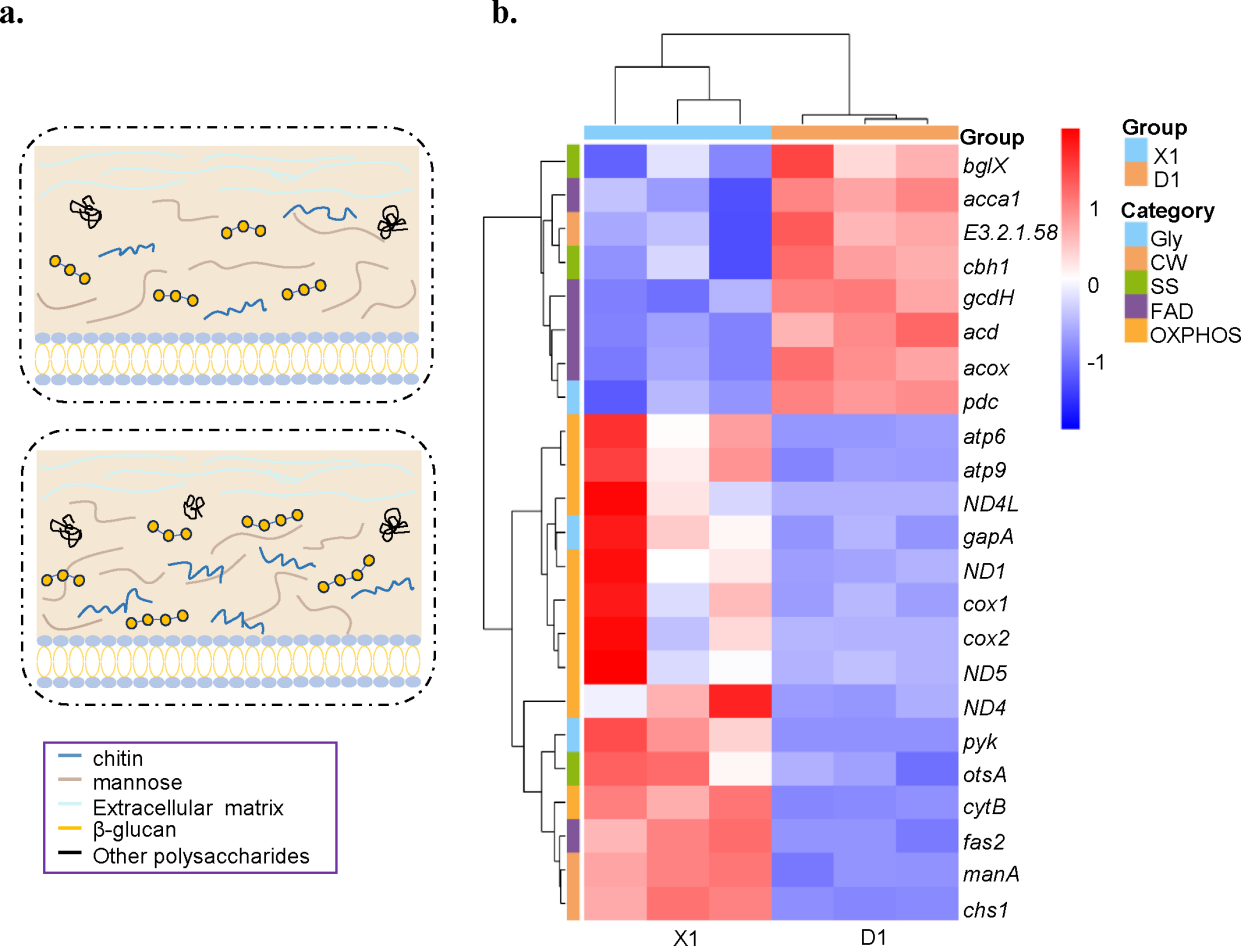
DEG overviews of *A. sydowii* after incubation for 3 days at different salt concentrations. (**a**) Diagram of the cell wall remodeling, top panel representing the cell wall remodeling under 0.5 M salinity, bottom panel representing the cell wall remodeling under 0.1 M salinity. (**b**) Heat map of marker genes analyzed in this study. Gly, glycolysis; CW, cell wall; SS, soluble substances; FAD, fatty acid degradation; OXPHOS, oxidative phosphorylation. X1 represents *A. sydowii* XTO612 cultured at 0.1 M salinity; X5 represents *A. sydowii* XTO612 cultured at 0.5 M salinity.

In our results, hypo-osmotic stress induced major changes in the transcriptional levels of DEGs related to cell wall components (Fig. 5a). Among these, the expression levels of the chitin synthase gene (*chs1*) and the mannose-6-phosphate isomerase gene (*manA*) were significantly upregulated. This suggests that the biosynthetic processes of chitin and mannose may be enhanced, leading to increased levels of these components in the cell wall. At the same time, the expression of the glucan 1,3-β-glucosidase (*EC 3.2.1.58*) gene was downregulated. The downregulation of this enzyme, which is mainly responsible for the hydrolysis of β-1,3-glucan, may result in the inhibition of β-1,3-glucan catabolism, which, in turn, may contribute to the accumulation of β-1,3-glucan in the cell wall. Next, a heat map containing the major genes associated with hyp-oosmotic stress tolerance was drawn (Fig. 5b).

### Energy metabolic pathways show variations under hypotonic stress

**Fig 6:**
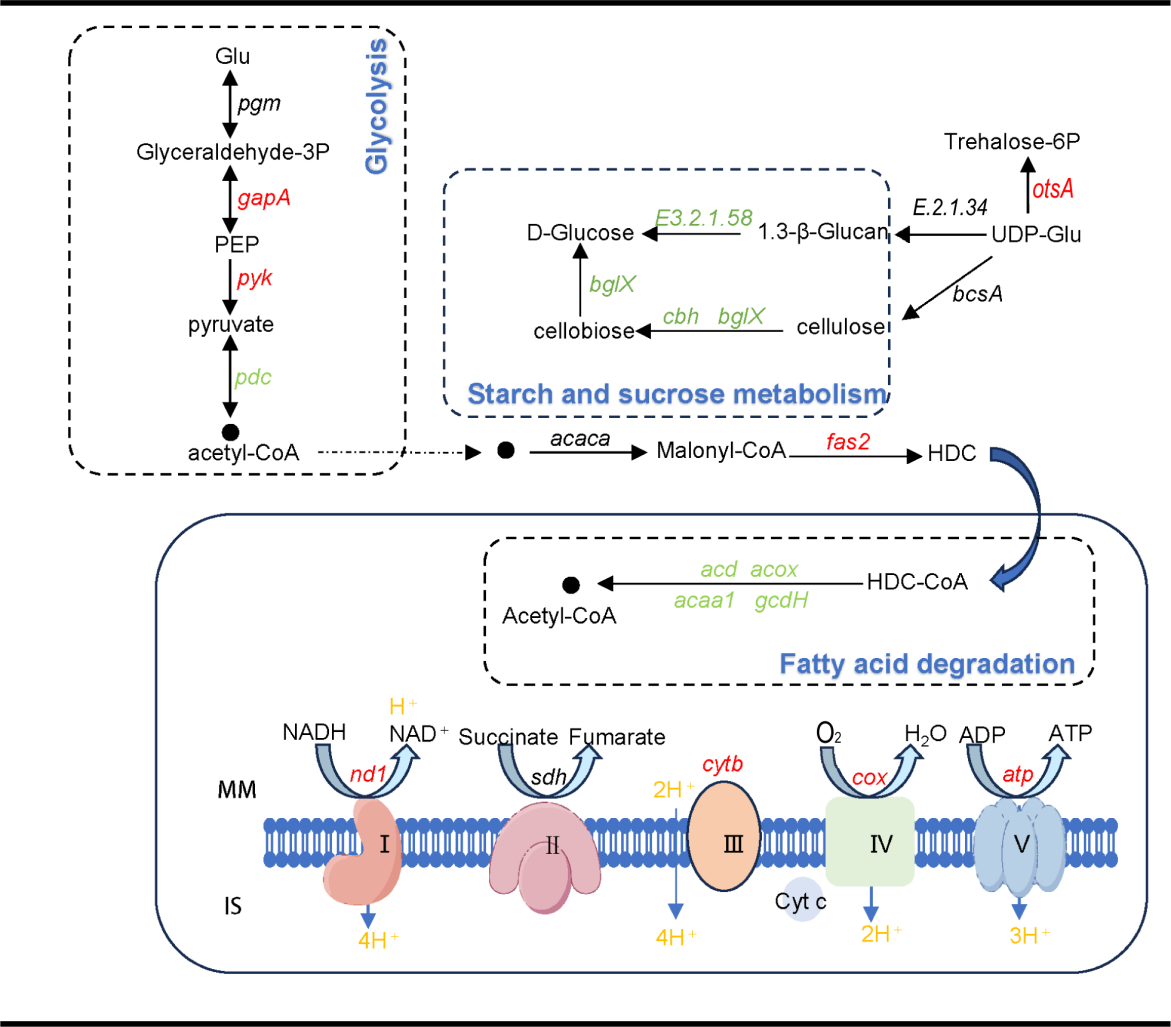
Energy metabolism pathway of *A. sydowii* XTO612 under low osmotic pressure conditions. Red-, green-, and black-labeled genes indicate that genes are upregulated, downregulated, and not significantly expressed, respectively. Gene names and the proteins they encode are listed in Table S4.

The expression of genes related to the oxidative phosphorylation pathway showed significant differences (Fig. 5b and 6). Eight genes were significantly upregulated, including the genes involved in the NADH-ubiquinone oxidoreductase chain (*ND4L, ND5, ND4,* and *ND1*), NADH: quinone reductase (*ndh*), ubiquinone-cytochrome c reductase cytochrome (*cytB*), and the F-type H + transporter ATPase subunit (*ATP9, ATP6*). At the same time, the expression of the cytochrome c oxidase subunit (*cox*) gene was downregulated. NAD+-dependent phosphorylation D-glyceraldehyde-3-phosphate dehydrogenase (*GAPDH, EC 1.2.1.12*) converts D-glyceraldehyde-3-phosphate to 1,3-diphosphoglycerate (50) and is a key enzyme in the glycolysis and gluconeogenesis pathways.

The expression of genes related to the glycolysis pathway showed significant differences (Fig. 5b and 6). The expression of two genes was upregulated, and one gene was downregulated. One of these is the upregulated expression of the pyruvate kinase (*pyk*) gene, which is a key regulator controlling metabolic fluxes and ATP production during glycolysis (51), while almost all *pyk* genes are heterologously regulated by various physiological effectors (52). Upregulation of the expression of the relevant genes is likely to increase the operational efficiency of the pathway, which, in turn, generates more ATP to adequately support the energy demands of the cells.

Differential expression of genes involved in the synthesis of soluble substances was also observed, which could contribute to the regulation of osmotic pressure (Fig. 5b and 6). The expression of the gene responsible for the synthesis of 6-phosphate alginate (*otsA*) was upregulated. This indicates an enhanced production of 6-phosphate alginate, which is a key component of alginate biosynthesis (53). On the other hand, the expression of the gene associated with sucrose degradation (*bglX*) was downregulated. This suggests a reduced capacity for sucrose degradation in the current conditions (54).

Fatty acid degradation, another pathway that generates acetyl-CoA, downregulation of four genes was also observed, including *acd*, *acox*, *acaa1*, and *gcdH,* which directly led to an increase in fatty acid content (Fig. 5b and 6). Another two genes associated with fatty acid synthesis subunit (*fas2*) and bifunctional Δ−12/ω-3 fatty acid desaturase (*K22993*) were upregulated significantly (log2 fold change ≥ 1, q value < 0.05) (Fig. 6; Table S4). The former implies an increased fatty acid content, while the latter is involved in the synthesis of unsaturated fatty acids. The function of these enzymes may increase the fluidity of the cell membrane, which was described in the extremely halotolerant black yeast (5). These adjustments may make *A. sydowii* XTO612 better adapted to low osmotic conditions.

## DISCUSSION

The primary distinction between freshwater and marine environments lies in the level of salinity (55, 56). Marine environments, characterized by high osmotic pressure, cause water loss from non-adapted cells (57). In response, marine microorganisms have evolved to restructure their life processes, enabling them to thrive in these hypertonic conditions. So, how do marine microbes respond when they are removed from the high osmotic pressure marine environment? In our work, a strain of *A. sydowii* XTO612 was isolated and characterized from the gut of a hadal amphipod. The physiological response to the osmotic stress was determined and compared with that of *A. sydowii* DM1, derived from hadal sediments. However, research on *A. sydowii* as a halophilic fungus has predominantly concentrated on adaptation mechanisms under high salinity conditions (30). Under hyperosmotic conditions, *A. sydowii* synthesizes glycerol, remodels cell wall architecture to enhance thickness, modifies membrane lipid composition, and upregulates hydrophobic protein biosynthesis (31). Under salt-free conditions, *A. sydowii* upregulates trehalose biosynthesis and hydrophobic protein-encoding genes. Concurrently, polyol accumulation correlates with constitutive activation of Hog1 kinase homologs in the absence of NaCl (30, 31). However, analogous investigations under low osmolarity conditions remain absent. Thus, here, low osmotic pressure was employed as an environmental stress to investigate the mechanism of *A. sydowii* response to osmotic stress in different habitats of the hadal. This should be the most comprehensive study of filamentous fungal growth under low osmotic pressure conditions in recent years and provides insights for future studies in environmental adaptation as well as in mechanisms that respond to low osmotic pressure.

There is limited information available on the adaptations and genomic information of cultivable filamentous fungi from different habitats under osmotic stress, particularly regarding their physiological properties and hypotonic stress relationships. This research novelly examines the divergent physiological responses of two *A. sydowii* strains to low osmotic pressure (0.1 M NaCl). *A. sydowii* from various sources exhibits high environmental adaptability. Comparing different isolates of the same fungal species can offer valuable insights into the environmental adaptation strategies, as demonstrated by *A. sydowii*’s pathogenicity to corals (11) and *Pseudomonas syringae*’s varying epiphytic competencies and hyperosmotic tolerance (12). This study compares the growth, development, and metabolism of two marine filamentous fungi at multiple phenotypic levels and employs transcriptomics to investigate their regulatory mechanisms underlying responses to environmental stress.

Recent research on marine fungi, especially *A. sydowii*, has made significant progress in drug discovery due to their production of chemically diverse compounds with broad-spectrum antibacterial, antiviral, and anticancer activities (9, 58, 59). It is well known that osmotic stress affects secondary metabolism in *Aspergillus* spp. (13). A recent study has linked VeA, a light-dependent global regulator controlling both sexual/asexual morphogenesis and secondary metabolism in filamentous fungi, to the osmotic-stress response in *A. flavus* (60). In our experiments, we found that the production of secondary metabolites by the fungus was promoted under low osmotic pressure conditions. While previous studies have shown that high osmolarity affects the production of fungal secondary metabolites, providing new directions for marine drug development, contrasts with previous studies that focused on high osmolarity effects. Future studies would focus on systematically comparing the specific compositional and structural differences of secondary metabolites produced by *A. sydowii* under varying osmotic conditions.

A notable finding from our study reveals that *A. sydowii* XTO612 lacked mycelial swelling under low osmolarity conditions, with reduced septal spacing compared to the optimal growth condition. While the function of mycelial septa in fungi remains unclear, previous studies suggest that they may enhance mycelial rigidity and provide structural support (61). Fungal cell walls are capable of remodeling their intrinsic components in response to external changes such as temperature, pH, osmotic stress, and other factors (62). Our transcriptome analysis revealed upregulation of genes related to cell wall remodeling, indicating enhanced capacity to synthesize unsaturated fatty acids and increase cell membrane fluidity. The divergent responses of the two *A. sydowii* strains to identical hypotonic stress can be attributed to differential environmental adaptation through evolutionary processes. The unique dietary structure of hadal amphipods, which creates a distinct gut environment different from the surrounding seawater and sediment, drives the microbial diversity within the gut (14). Such dynamic gut microenvironments may drive microbial evolutionary adaptations, enabling *A. sydowii* XTO612 to develop enhanced regulatory capacity for rapid osmotic stress response. Under hypotonic conditions, *A. sydowii* XTO612 may employ a comprehensive regulatory strategy—potentially involving modulation of membrane permeability, cell wall remodeling, energy metabolism, and osmolyte biosynthesis pathways—to optimize osmotic homeostasis, which could provide ecological value in its host, through enhanced osmotic adjustment mechanisms (31). However, further experimental validation is required to substantiate these proposed mechanisms.

Last but not least, our work provides new insights into the adaptation of filamentous fungi to low osmotic pressure environments. However, we recognize that there is still a lack of further exploration into *A. sydowii*’s biotechnological potential. For instance, the potential applications of these metabolic responses in bioengineering and pharmaceutical development have not been fully explored. Additionally, simulating its *in situ* environment is challenging due to significant differences between laboratory conditions and natural habitats, which may limit our comprehensive understanding of its ecological adaptation mechanisms. Although this work represents only a small aspect of the study of the environmental adaptation of marine filamentous fungi, it is an important step toward understanding the mechanism of their osmotic pressure adaptation.

## Data Availability

The entire *ITS*, *benA*, and *cam* genes in *A. sydowii* XTO612 were submitted to the NCBI, accession numbers PV412627 (*ITS*), PV459640 (*cam*), and PV459641 (*benA*). Raw sequencing reads for the transcript information in this work were submitted to the NCBI Sequence Read Archive under sequential accession numbers from SAMN47618251 to SAMN47618262. All data have been made public in the NODE database (National Omics Data Encyclopedia), including the gene sequences OEZ00021680 (*ITS*), OEZ00021681 (*benA*), and OEZ00021682 (*cam*), as well as the raw sequencing reads of the transcriptome OEX00032685. The NODE database can be accessed via the website https://www.biosino.org/node/home. Correspondence and requests for materials should be addressed to Xi Yu.

## SUPPLEMENTAL MATERIAL

Supplemental material aem.01448-25-s0001.docx Figures S1 to S5; Tables S1 to S3.

Table S4 aem.01448-25-s0002.xlsx Summary of differential genes in two strains of *A. sydowii* under hypotonic conditions.

ASM does not own the copyrights to Supplemental Material that may be linked to, or accessed through, an article. The authors have granted ASM a non-exclusive, world-wide license to publish the Supplemental Material files. Please contact the corresponding author directly for reuse.
