# Supplementary material for: Differential hypo-osmotic stress responses and regulatory mechanisms of Aspergillus sydowii in amphipod guts and hadal sediments
Source: Appl Environ Microbiol. 2025 Oct 29;91(11):e01448-25. doi: 10.1128/aem.01448-25 (PMC12628754; doi:10.1128/aem.01448-25)
Supplement: Supplemental material — Figures S1 to S5; Tables S1 to S3. [file aem.01448-25-s0001.docx]

**Differential Hypo-osmotic Stress Responses and Regulatory Mechanisms of *Aspergillus sydowii* in Amphipod Guts and Hadal Sediments**

Zhuo Wang^1^, Yukun Cui^1^, Jiasong Fang^1^ and Xi Yu^1^*

^1^ Shanghai Engineering Research Center of Hadal Science and Technology, College of Oceanography and Ecological Science, Shanghai Ocean University, Shanghai, 201306, China

*Corresponding author: XY (x[yu@shou.edu.cn](mailto:yu@shou.edu.cn)) ORCID: http://orcid.org/0000-0003-4243-6016

**Running head**: Hypo-osmotic Stress in Fungi

**Key words**: hadal amphipods; *Aspergillus sydowii*; transcriptomic analyses; osmotic stress; regulation mechanism


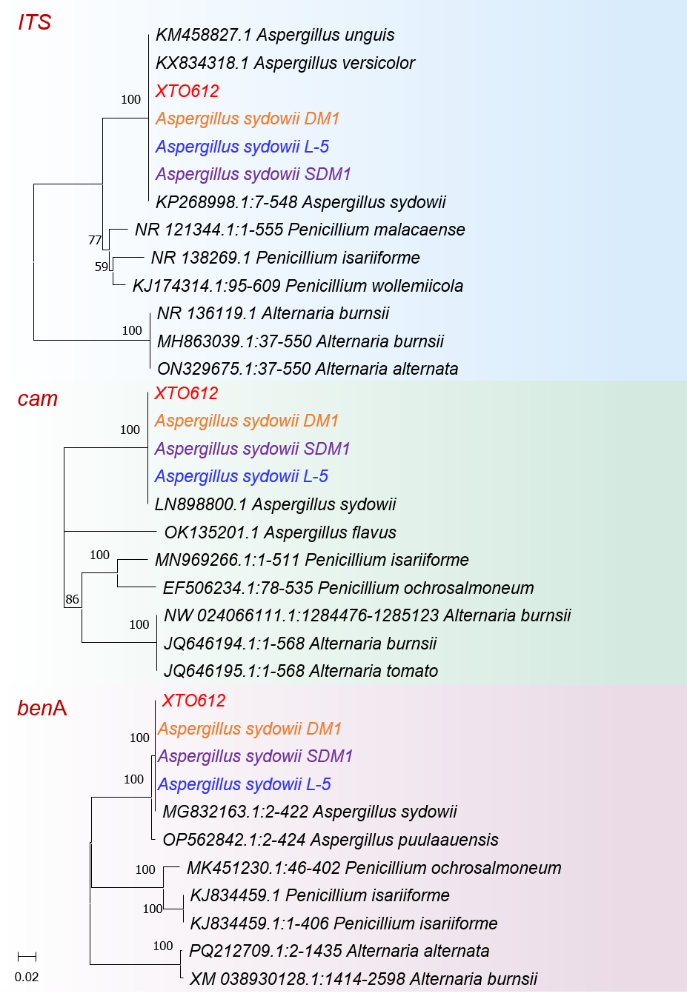

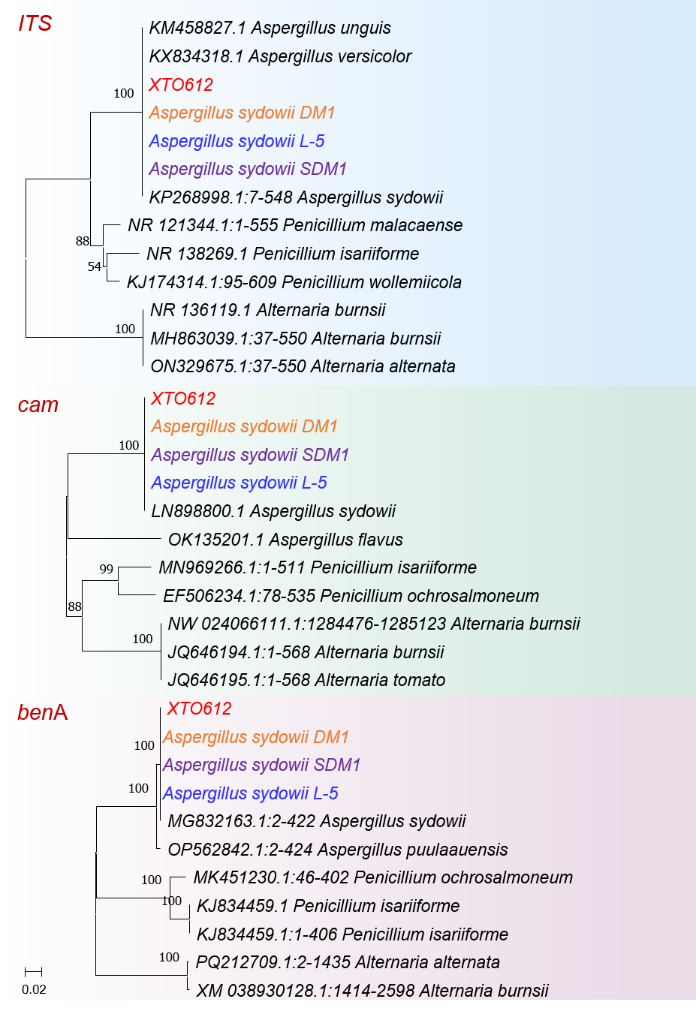
a. b.

**Fig S1** Identification of different sources of *A. sydowii*. (a) Phylogenies were reconstructed for the *ITS*, *cam*, and *benA* gene sequences inde-pendently using Neighbor-Joining. (b) Phylogenies were reconstructed for the *ITS*, *cam*, and *benA* gene sequences inde-pendently using Maximum parsimony. Bootstrap analysis was performed using 1,000 replicates.


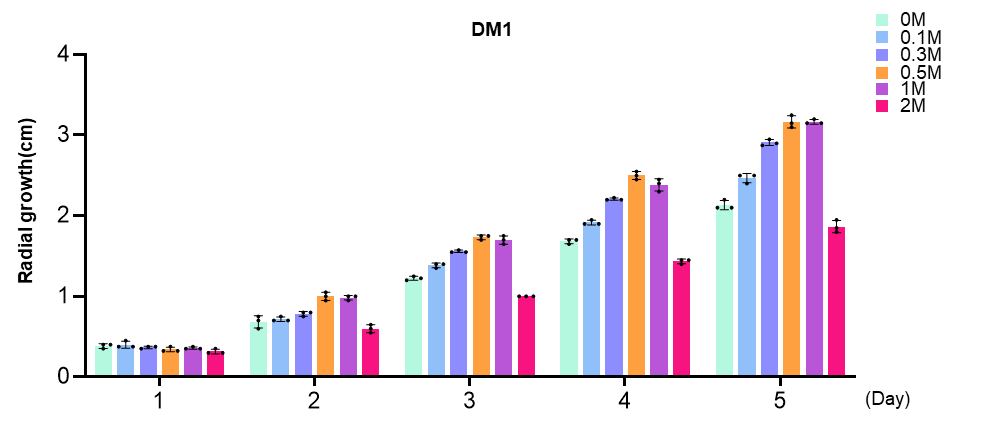

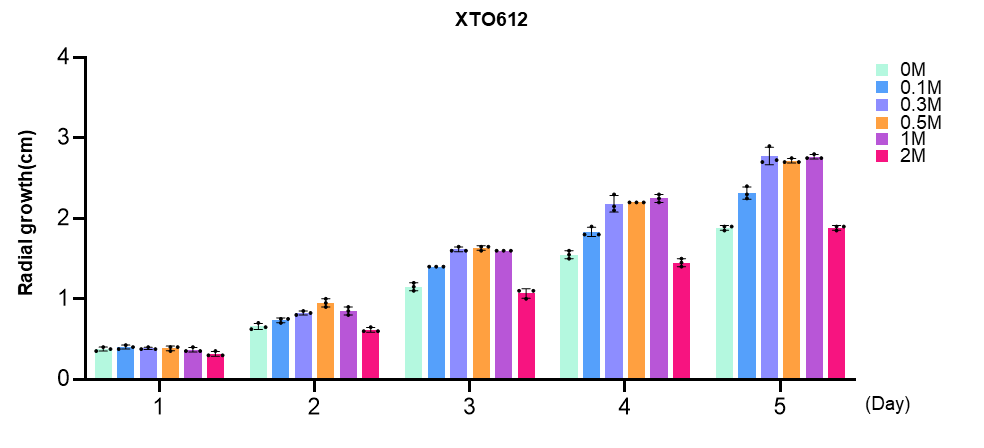
a.

**Fig S2** Growth rate of *A. sydowii* from different sources on PDA within 5 days. (a) *A. sydowii* XTO612. (b) *A. sydowii* DM1. Data are means of three replicates. *A. sydowii* incubated in 0 M NaCl was used as control. Error bars in the bar graph represent the standard deviation of the mean. The picture was plotted in GraphPad Prism 8.

b.

**
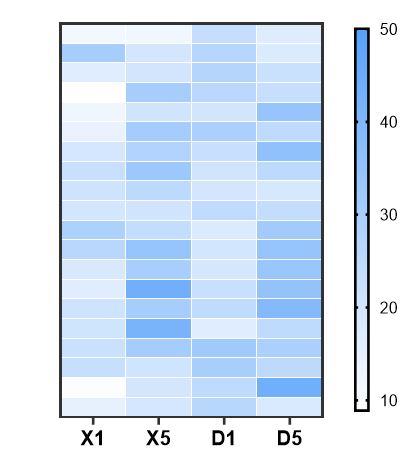
**

**Fig S3** Septal lengths of two *A. sydowii* strains under 0.1 M and 0.5 M salinity conditions. The picture was plotted in GraphPad Prism 8. X1 represents *A. sydowii* XTO612 cultured at 0.1 M salinity; X5 represents *A. sydowii* XTO612 cultured at 0.5 M salinity; D1 represents *A. sydowii* DM1 cultured at 0.1 M salinity; D5 represents *A. sydowii* DM1 cultured at 0.5 M salinity.


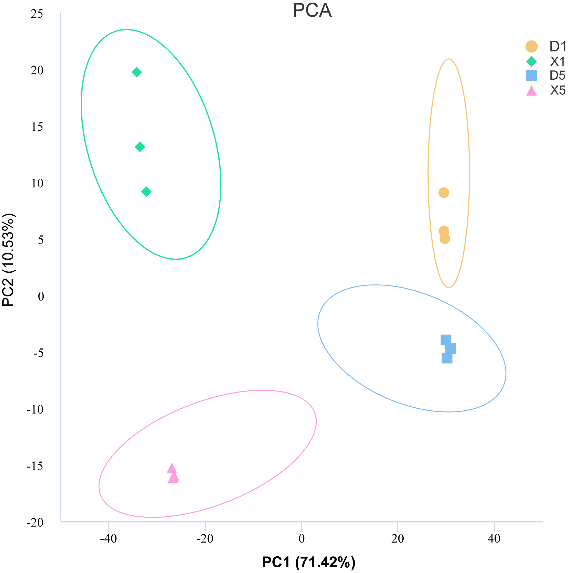
a.


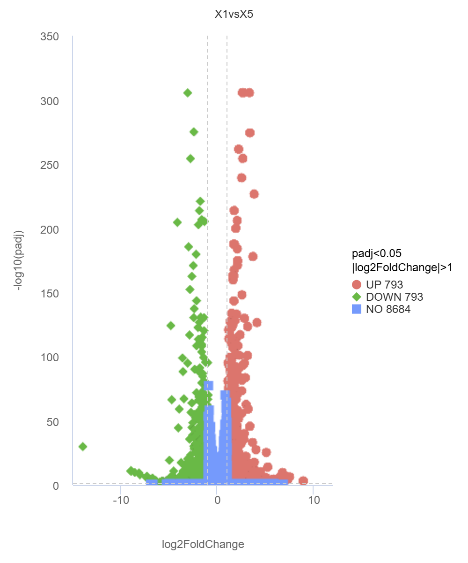

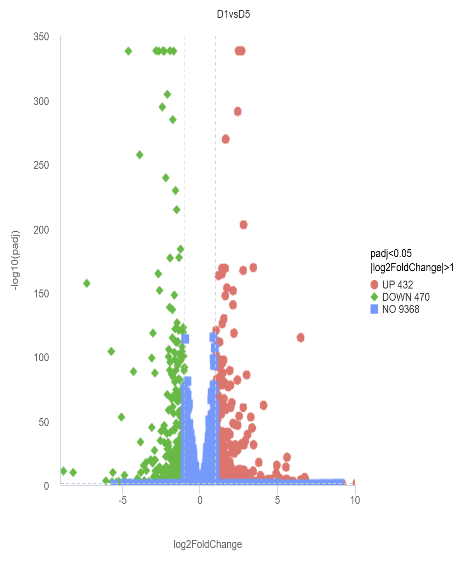
b. c.

**Fig S4** Transcriptome overview. (a) Principal component analysis (PCA) of the gene expression profiles in different groups. Princomp function in RStudio 2022.4.2.0 performs PCA analysis and figure was were drawn using ggplot2 package. (b-c) The number of differentially expressed genes (log2 foldchange > 1 or <-1) is shown in the volcano plot. (b) The volcano plot was analyzed using R (Version 3.0.3) ggplot2 package. A total of 10270 DEGs, including 793 up-regulated genes and 793 down-regulated genes, were detected in *A. sydowii* XTO612 cultured at 0.1 M salinity compared to 0.5 M. (c) A total of 902 DEGs, including 432 up-regulated genes and 470 down-regulated genes, were detected in *A. sydowii* DM1 cultured at 0.1 M salinity compared to 0.5 M.


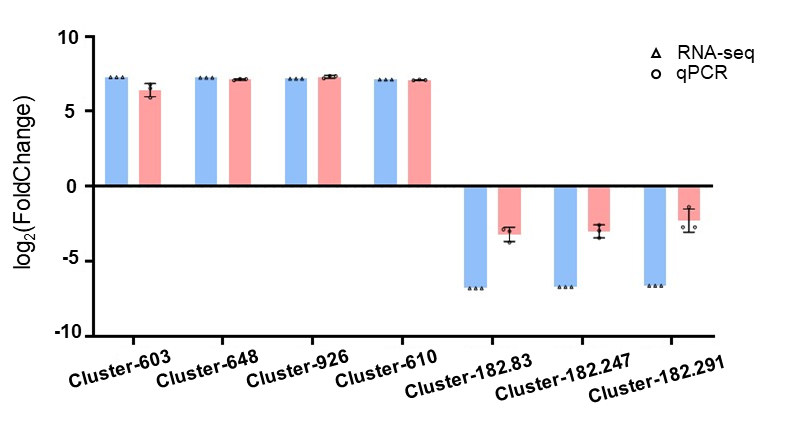
a.


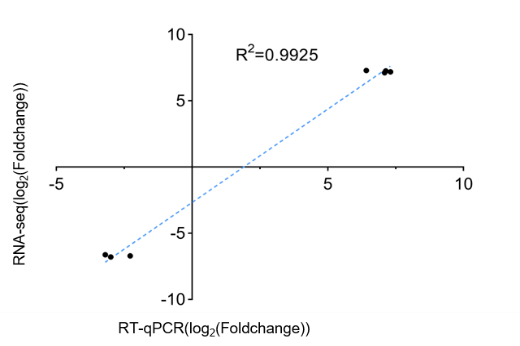
b.

**Figure S5** Expression levels of differently expressed genes validated by RT-qPCR. (a) Expression levels of differently expressed genes validated by qRT-PCR. (b) The value correlations between RNA-seq and qRT-PCR under 0.1 M NaCl condition of *A. sydowii* XTO612. R^2^ indicates the R-square of the regression line. The correlations between RNA-Seq and qRT-PCR exhibited well (R^2^ > 0.9). Error bars in column graphs represent standard deviation of the mean. The picture was plotted in GraphPad Prism 8.

**Table S1** Primers information used in this work.

| **Gene/Gene ID** | **Primer name** | | | **sequence (5'-3')** | **Purpose** |
| --- | --- | --- | --- | --- | --- |
| *ITS* | | ITS1 | TCCGTAGGTGAACCTGCGG | | phylogeny marker |
|  |  | ITS4 | TCCTCCGCTTATTGATATGC | |  |
| *ben*A | | Bt2a | GGTAACCAAATCGGTGCTGCTTTC | | phylogeny marker/reference gene |
|  |  | Bt2b | ACCCTCAGTGTAGTGACCCTTGGC | |  |
| *cam* | | CMD5 | CCGAGTACAAGGARGCCTTC | | phylogeny marker |
|  |  | CMD6 | CCGATRGAGGTCATRACGTGG | |  |
| *Cluster-603* | | 603F1 | ACTTCTTCTTCCTACAGGTTGA | | RT-qPCR |
|  |  | 603R1 | GCTTAGTTATGATTGCCTCTCA | |  |
| *Cluster-648* | | 648F1 | AGTGTTGATGCCAATATGAGGT | | RT-qPCR |
|  |  | 648R1 | TCAATCAGTACGCCATAGTGTT | |  |
| *Cluster-926* | | 926F1 | CGTGCCATTGATGCAGAGG | | RT-qPCR |
|  |  | 926R1 | AACCATTGCTACCTTCGTTGAA | |  |
| *Cluster-610* | | 610F1 | AGCGTTTCTCACTTGTTAAGGT | | RT-qPCR |
|  |  | 610R1 | CGTTGACAACATGCCGACAA | |  |
| *Cluster-182.83* | | 182.83F1 | CCTGATGAGAAGACCGTGTTC | | RT-qPCR |
|  |  | 182.83R1 | CATTGACTGTGATCCGCTGAG | |  |
| *Cluster-182.247* | | 182.247F1 | TGCTCAACATCGGCGTAATC | | RT-qPCR |
|  |  | 182.247R1 | GGCGAAGGAGAAGGAGAAGT | |  |
| *Cluster-182.291* | | 182.291F1 | CCTATGGCTCGGCTCTGTT | | RT-qPCR |
|  |  | 182.291R1 | CAGTGTCTTCGTCGCATCC | |  |

**Table S2** UPLC-MS/MS diagram of secondary metabolites of *A. sydowii*. a. The top 10 compounds in group D1 vs X1. D1 represents *A. sydowii* DM1 cultured at 0.1 M salinity; X1 represents *A. sydowii* XTO612 cultured at 0.1 M salinity.

| Spectrum ID | Compound Name | Formula | SpecMZ | Structure |
| --- | --- | --- | --- | --- |
| A-1 | Unguisin B | C_37_H_56_N_8_O_7_ | 723.4173 | 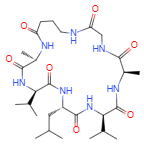 |
| A-2 | Aspergillicin E | C_39_H_58_N_6_O_9_ | 753.4199 | 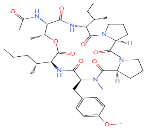 |
| A-3 | Radarin B | C_28_H_41_NO_2_ | 422.3045 | 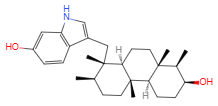 |
| A-4 | Asperorydine N | C_15_H_18_N_2_O_3_ | 273.1246 | 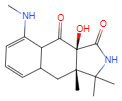 |
| A-5 | Cosmosporaside C | C_34_H_60_O_15_ | 707.3861 | 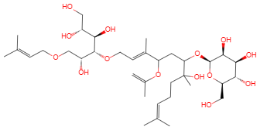 |
| A-6 | Hormonemate A | C_35_H_62_O_15_ | 721.4016 | 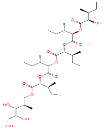 |
| A-7 | 2-(ethoxycarbonyl)-4'-carboxydiorcinal | C_18_H_18_O_7_ | 345.0979 | 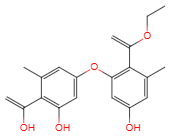 |
| A-8 | Albatrelin F | C_44_H_58_O_6_ | 681.4194 | 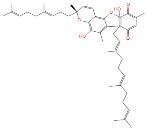 |
| A-9 | 18-O-b-d-Glucopyranosyl-18S-hydroxyneodihydroprotolichesterinate21-O-b-d-glucopyranoside | C_33_H_58_O_15_ | 693.3703 | 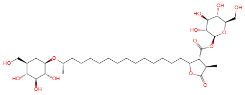 |
| A-10 | Calpinactam | C_38_H_57_N_9_O_8_ | 766.4232 | 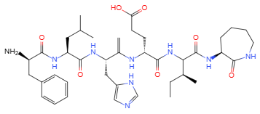 |

**Table S3** Statistical summary of the results of RNA-seq data. (D1 represents *A. sydowii* DM1 cultured under 0.1 M NaCl; D5 represents *A. sydowii* DM1 cultured under 0.5 M NaCl; X1 represents *A. sydowii* XTO612 cultured under 0.1M NaCl; X5 represents *A. sydowii* XTO612 cultured under 0.5 M NaCl).

| Sample name | total raw  reads(M) | total clean  reads(M) | total clean  bases | clean read Q20(%) | clean read Q30(%) | clean read ratio(%) |
| --- | --- | --- | --- | --- | --- | --- |
| D1_1 | 23.22 | 22.46 | 6.74 | 98.43 | 95.54 | 96.73 |
| D1_2 | 23.92 | 23.46 | 7.04 | 98.74 | 96.3 | 98.08 |
| D1_3 | 23.21 | 22.56 | 6.77 | 98.6 | 96.03 | 97.20 |
| D5_1 | 23.21 | 22.57 | 6.77 | 98.63 | 96.11 | 97.24 |
| D5_2 | 23.55 | 23.22 | 6.97 | 98.66 | 96.16 | 98.60 |
| D5_3 | 23.57 | 22.81 | 6.84 | 98.58 | 95.96 | 96.78 |
| X1_1 | 24.04 | 23.07 | 6.92 | 98.5 | 95.98 | 95.97 |
| X1_2 | 26.58 | 25.6 | 7.68 | 98.52 | 95.94 | 96.31 |
| X1_3 | 22.85 | 22.03 | 6.61 | 98.41 | 95.39 | 96.41 |
| X5_1 | 22.97 | 22.32 | 6.7 | 98.7 | 96.26 | 97.17 |
| X5_2 | 22.71 | 21.87 | 6.56 | 98.73 | 96.31 | 96.30 |
| X5_3 | 22.80 | 22.05 | 6.62 | 98.76 | 96.4 | 96.71 |
